# Impact of Early Colonizers on *In Vitro* Subgingival Biofilm Formation

**DOI:** 10.1371/journal.pone.0083090

**Published:** 2013-12-05

**Authors:** Thomas W. Ammann, Georgios N. Belibasakis, Thomas Thurnheer

**Affiliations:** Oral Microbiology and Immunology, Institute of Oral Biology, Center of Dental Medicine, University of Zürich, Zürich, Switzerland; University Hospital of the Albert-Ludwigs-University Freiburg, Germany

## Abstract

The aim of this study was to investigate the impact of early colonizing species on the structure and the composition of the bacterial community developing in a subgingival 10-species biofilm model system. The model included *Streptococcus oralis*, *Streptococcus anginosus*, *Actinomycesoris*, *Fusobacterium nucleatum* subsp. *nucleatum*, *Veillonella dispar*, *Campylobacter rectus*, *Prevotella intermedia*, *Porphyromonas gingivalis*, *Tannerella forsythia*, and *Treponema denticola*. Based on literature, we considered *Streptococcus oralis*, *Streptococcus anginosus*, and *Actinomyces oris* as early colonizers and examined their role in the biofilms by either a delayed addition to the consortium, or by not inoculating at all the biofilms with these species. We quantitatively evaluated the resulting biofilms by real-time quantitative PCR and further compared the structures using confocal laser scanning microscopy following fluorescence *in situ* hybridisation. The absence of the early colonizers did not hinder biofilm formation. The biofilms reached the same total counts and developed to normal thickness. However, quantitative shifts in the abundances of individual species were observed. In the absence of streptococci, the overall biofilm structure appeared looser and more dispersed. Moreover, besides a significant increase of *P. intermedia* and a decrease of *P. gingivalis* , *P. intermedia* appeared to form filamented long chains that resembled streptococci. *A. oris*, although growing to significantly higher abundance in absence of streptococci, did not have a visible impact on the biofilms. Hence, in the absence of the early colonizers, there is a pronounced effect on *P. intermedia* and *P. gingivalis* that may cause distinct shifts in the structure of the biofilm. Streptococci possibly facilitate the establishment of *P. gingivalis* into subgingival biofilms, while in their absence *P. intermedia* became more dominant and forms elongated chains.

## Introduction

Periodontitis is one of the most prevalent diseases worldwide. Based on the evaluation of the NHANES 2009-2010, Eke et al. [1] stated the prevalence of periodontitis in the United States in adults aged 30 years and older to 47.2 %. In adults older than 65 years, the prevalence is even higher with 70.1 %. In 1999, the costs for treatment and prevention of the disease reached 14.3 billion dollars [2]. Despite the prevalence and costs caused by the disease, knowledge is still very limited. Key factor for this is the complex nature of periodontitis [3]. There is no single causative organism for the disease, but the interplay of a consortium of several hundred species, which as well do vary significantly between patients and sites [4]. Even though there have been large efforts to bring light to the pathogenicity during the past decades, many mechanisms involved in the aetiology of periodontitis still remain unclear.

The constant bacterial colonization and growth on the tooth surfaces leads to the formation of oral biofilms, which can only be controlled by daily oral hygiene. With the abstinence of oral hygiene, as is the case in the experimental gingivitis in man, the bacterial composition of these biofilms, initially dominated by cocci and small rods, starts to shift towards a spirochaete-dominated flora, accompanied by the onset of gingivitis over a period of two to three weeks [5]. This transition of the bacterial flora seems to be the key process in the induction of periodontitis at a later stage. However, how this succession of species is guided is still unclear. Species associated with different stages periodontal disease were defined more than 10 years ago [6,7] and, more recently, the normal bacterial flora in the oral cavity as well [8]. While *Streptococcus sp.* and *Actinomyces sp.* are recognized as dominant species in the healthy oral flora and their role as early colonizers is understudied. They were observed *in vivo* as the bacteria attaching directly to the tooth surface [9], and in terms of attachment capabilities they are the ones that are able to directly bind to the salivary pellicle [10]. However, the sequence of events responsible the changes from biofilms dominated by these early colonizers, to the completely altered consortium detected in periodontal pockets is still being debated. It was speculated that the generally symbiotic oral community is modified by certain keystone pathogens that are, even at low abundance, capable to impair the host’s immune response and increase the pathogenic potential of the whole community [11].

In this study, we used a 10-species subgingival biofilm model system to address the question how the early colonizing species, namely *Streptococcus oralis*, *Streptococcus anginosus*, and *Actinomyces oris* influence the development of the biofilms. While the full consortium of these 10 species was shown to produce biofilms resembling a state of chronic periodontitis in both quantitative distribution of the bacteria and structure [12], we hypothesized that the removal of these species will either significantly hinder biofilm formation or reduce the quantity of later colonizing species. We used quantitative real-time PCR (qPCR) and confocal laser scanning microscopy (CLSM) following fluorescence in situ hybridisation (FISH) to compare the quantitative distribution of the bacteria and the three dimensional structure of biofilms originating from inocula either without early colonizing species or with early colonizers added at a later time point.

## Materials and Methods

### Biofilm cultivation

Ten bacterial strains were used for the cultivation of the model biofilms: *Streptococcus oralis* SK248 (OMZ 607), *Streptococcus anginosus* ATCC 9895 (OMZ 871), *Actinomyces oris* (OMZ 745), *Fusobacterium nucleatum* subsp. *nucleatum*OMZ 598, *Veillonella dispar* ATCC 17748^T^ (OMZ 493), *Campylobacter rectus* OMZ 698, *Prevotella intermedia* ATCC 25611^T^ (OMZ 278), *Porphyromonas gingivalis* ATCC 33277^T^ (OMZ 925), *Tannerella forsythia* OMZ 1047, and *Treponema denticola* ATCC 35405^T^ (OMZ 661).

Four different biofilm types were cultured from four different inocula (in the following also designated as four different approaches). The cultivation of the biofilms followed the methodology described before [12], with the exception of the changes described in the following. In short, all strains were precultured in adequate liquid media to reach late exponential growth prior to adjusting the optical density at 550 nm to 1.0 for all strains except *T. denticola* and *C. rectus*, which were set to an optical density of 0.5. Before the inoculation, the sintered hydroxy apatite discs were allowed to acquire a salivary pellicle. The discs were placed in 24-well plates (one per well) and covered with 800 µl of saliva, diluted 1:2 with a mixture of 0.9 % NaCl and distilled water and incubated for 4 h on a rotary shaker at 90 RPM. Then the discs were placed in fresh in 24-well plates (one per well), covered with 1.5 ml of biofilm growth medium and inoculated with 200 μl of inoculum mixture. The growth medium was composed of 50 % heat inactivated horse serum and 50 % of modified fluid universal medium (“mFUM”; [13]). To promote growth of *T. forsythia*, N-acetylmuramic acid at a final concentration of 0.34 mM was added [14] and to optimise growth of *P. gingivalis*, haemin at a final concentration of 15.3 µM was added [15].

Four different inocula were used in this study: As a control, an inoculum containing all 10 species was used. The inoculum “LateStr”, (“late streptococci”) harboured all ten species, however, the two streptococcus strains were added after 16.5 h of incubation time. “NoStr”, (“no streptococci”) used no streptococci at all, and in “NoStrNoAori” (“no streptococci, no *A. oris*”), neither the streptococci, nor *A. oris* were included in the inoculum. 

The incubation time for the biofilms was 64.5 h. The growth medium was renewed first after 16.5 h and subsequently every 24 h. In addition, the biofilms were dip-washed in 0.9 % NaCl twice daily (morning and afternoon) at an interval of 6 h. The morning dips were performed prior to each renewal of the growth medium or, at the end of the incubation time, prior to the harvest of the biofilms.

### qPCR

The conditions and primers used for the qPCR were the same as described before [16]; with the only difference that a five times higher concentration of DNA per reaction was used for the qPCR to ensure no strains falling below the detection limit. Three discs per experiment and approach were processed for qPCR quantification.

Biofilms were removed from the hydroxyapatite discs by vortexing in 0.9 % NaCl, followed by low-power sonication to better disperse the cells. From the obtained bacterial mixtures, DNA was extracted using the GenElute Bacterial Genomic DNA Kit (Sigma). The extraction was performed according to the manufacturer’s guidelines, however, with the incubation times for the two lysis steps doubled. The amount of extracted DNA from biofilms (as well as the DNA concentration for the standard curves) was determined using a NanoDrop ND-1000 device (Thermo-Fisher Scientific).

Ten pairs of specific primers, one for each species, were used for the qPCR. Reactions were run in doubles and with two dilutions (5 ng and 0.5 ng total DNA per reaction) for each species and sample. The amount of DNA for each species was calculated using C_q_ vs. log (DNA [ng]) standard curves, and the actual cell numbers were calculated based on the theoretical genome weight of each organism and the amount of DNA determined by the qPCR.

### FISH

The 16S rRNA probes and the general working procedure for the FISH staining were the same as described before [12], however, the incubation time for the hybridisation of the probes was extended from 3 h to overnight. In two experiments, five intact biofilms per approach were processed for FISH staining and subsequently analysed by CLSM.

Following the last dip-washing, mature 64.5 h biofilms were fixed *in situ* attached to the hydroxyapatite discs in 4 % paraformaldehyde solution for at least 3 h in the dark at 4 °C. After 15 min of pre-hybridisation in hybridisation buffer alone, the biofilms were transferred to the hybridisation buffer containing a selected mixture of two 16S rRNA probes. Thus, each biofilm was stained specifically for two species, using one Cy3 and one Cy5 labelled probe. After overnight incubation, biofilms were washed in washing buffer and counterstained using a combination of the DNA stains SYTOX Green (Invitrogen) and Yo-Pro-1 iodide (Invitrogen). After the staining procedure, biofilms were embedded upside down on chamber slides in MOWIOL [17].

### CLSM and Image Analysis

All images from FISH stained biofilms were taken at a Leica SP-5 CLSM provided by the Center of Microscopy and Image Analysis of the University of Zürich (ZMB). Images were recorded on three channels simultaneously at the frequency of 8000 Hz using the resonant scanner available with the system. Biofilms were carefully screened to determine if biofilms a) were intact, b) had similar thickness throughout the whole disc area, and c) showed a repetitive distribution pattern of the stained bacteria. If these criteria were met, a representative area was selected and a stacked image was recorded. 

Stacked images were further processed using Imaris 7.4.0 (Bitplane). Snapshots presented in this paper were additionally sharpened using the GIMP version 2.6 (http://www.gimp.org).

### Statistics

The presented data are from three independent experiments. For quantification, biofilms from each approach (i.e. each inoculum) were cultivated in triplicates, resulting in N=9 biofilms per approach.

For the generation of the box-plots and the statistical calculations, SPSS Statistics 20 (IBM Software) was used. To test for significant differences between cell numbers detected with the different approaches the data were logarithmically transformed prior to the analysis by one-way ANOVA, with the Bonferroni correction for multiple comparisons.

## Results

### Quantification

**Figure 1:**
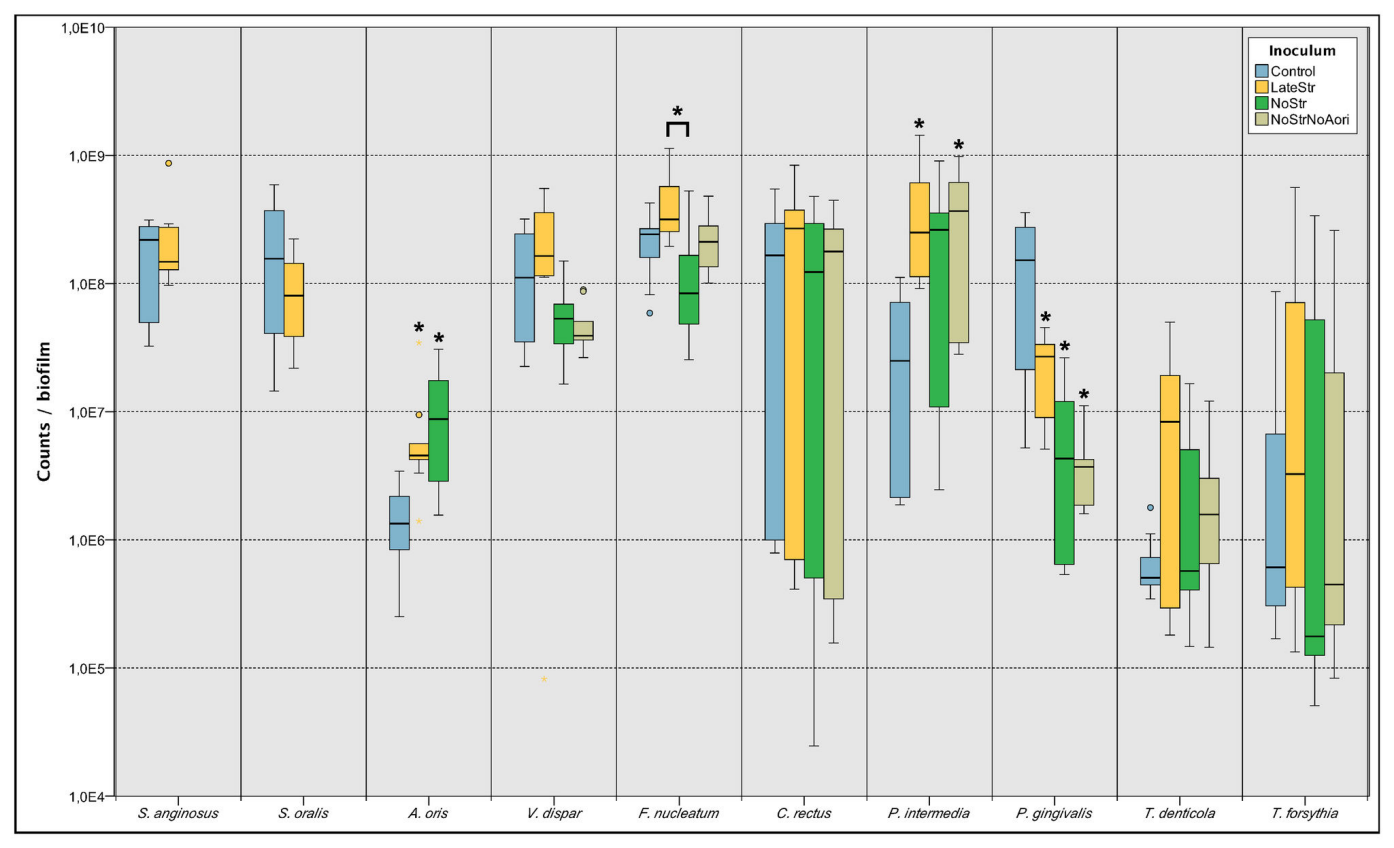
Species-specific cell numbers in the biofilms after 64.5 h of incubation. The boxes show data from three independent experiments, each performed with triplicate biofilms. The different colours indicate the type of inoculum. Control: All ten species. LateStr: Inoculation without streptococci, addition of streptococci after 16.5 h. NoStr: No streptococci. NoStrNoAori: No streptococci, no *A*. *oris*. The boxes represent the inter-quartile range of the data points, the bar indicates the median. The whiskers cover the data points within the 1.5x inter quartile range. * indicates a significant difference (p ≤ 0.05) between the indicated box and the control, or between the boxes indicated by brackets.

**Figure 2:**
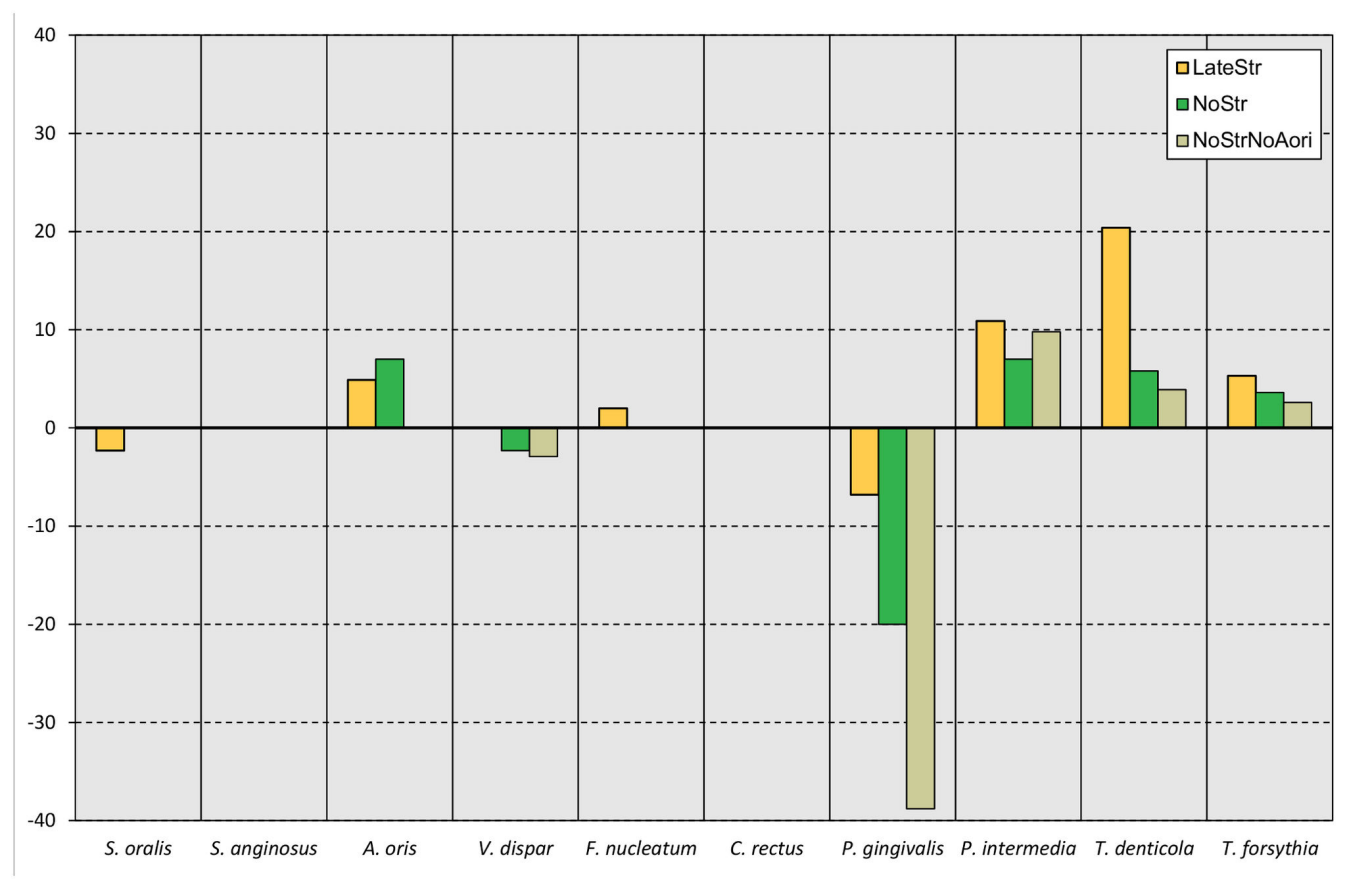
Individual inoculation specific shifts in abundances of the species in comparison to control biofilms inoculated with all ten species after 64.5 h of incubation. The calculation is based on the average values of each species and inoculum type (N=9). Control: All ten species. LateStr: Inoculation without streptococci, addition of streptococci after 16.5 h. NoStr: No streptococci. NoStrNoAori: No streptococci, no *A*. *oris*.

The numbers of the individual bacterial species in each biofilm group were firstly determined. Irrespective of the inoculum the 64.5 h biofilms did not show significant differences in total counts (Figure S1). However, with regard to the abundances of the individual species, significant inoculum-dependent shifts within the bacterial community were observed concerning four out of the 10 species used in the biofilm model (Figure 1). For better visualisation, Figure 2 summarises the changes in abundances for all species that reached a level greater than 2-fold in comparison to control biofilms. In particular, the addition of the streptococci to the consortium after 16.5 h produced significantly higher cell numbers of *A. oris* and *P. intermedia*, while *P. gingivalis* counts were significantly reduced. The omission of streptococci led to a significant increase of the abundance of *A. oris*, while it significantly decreased that of *P. gingivalis*. Removing *S. oralis*, *S.* anginosus, and *A. oris* from the inoculum resulted in a significant increase of *P. intermedia* counts together with a significant decrease of *P. gingivalis* counts. Further, *F. nucleatum* showed significantly improved growth following the late addition of the streptococci, in comparison to the approach with the complete absence of streptococci. However, both these values did not reach significance if compared to the control 10-species biofilms.

### Structure

**Figure 3:**
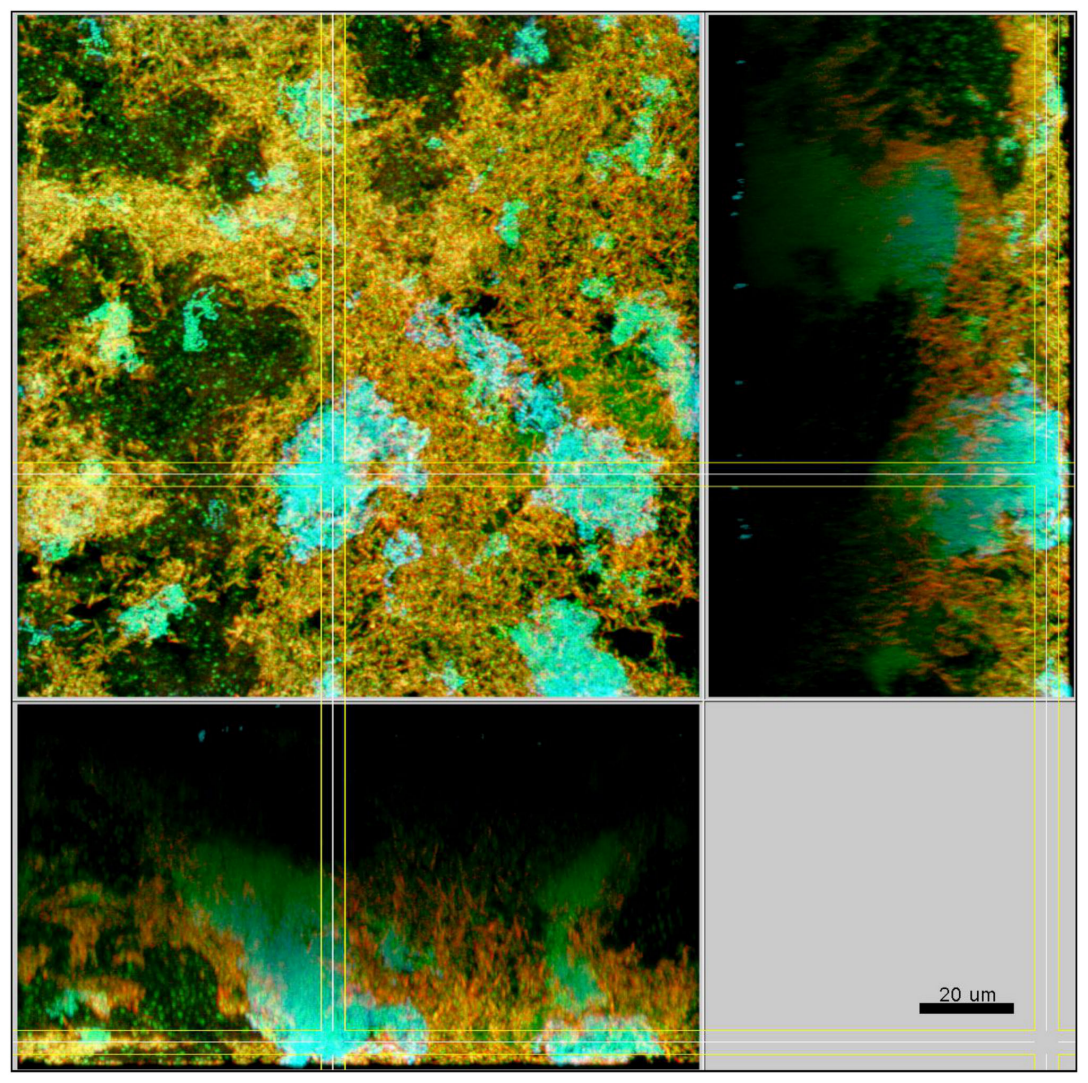
Structure of a control 10-species biofilm after 64.5 h of incubation. Yellow / red: *F*. *nucleatum*, cyan: streptococci, green: non-hybridized cells (DNA-staining Yo-Pro-1 + Sytox Green). The yellow colour of *F*. *nucleatum* is due to the cross-staining of the Fnuc133c-Cy3 probe (red) and the universal DNA stains (green).

**Figure 4:**
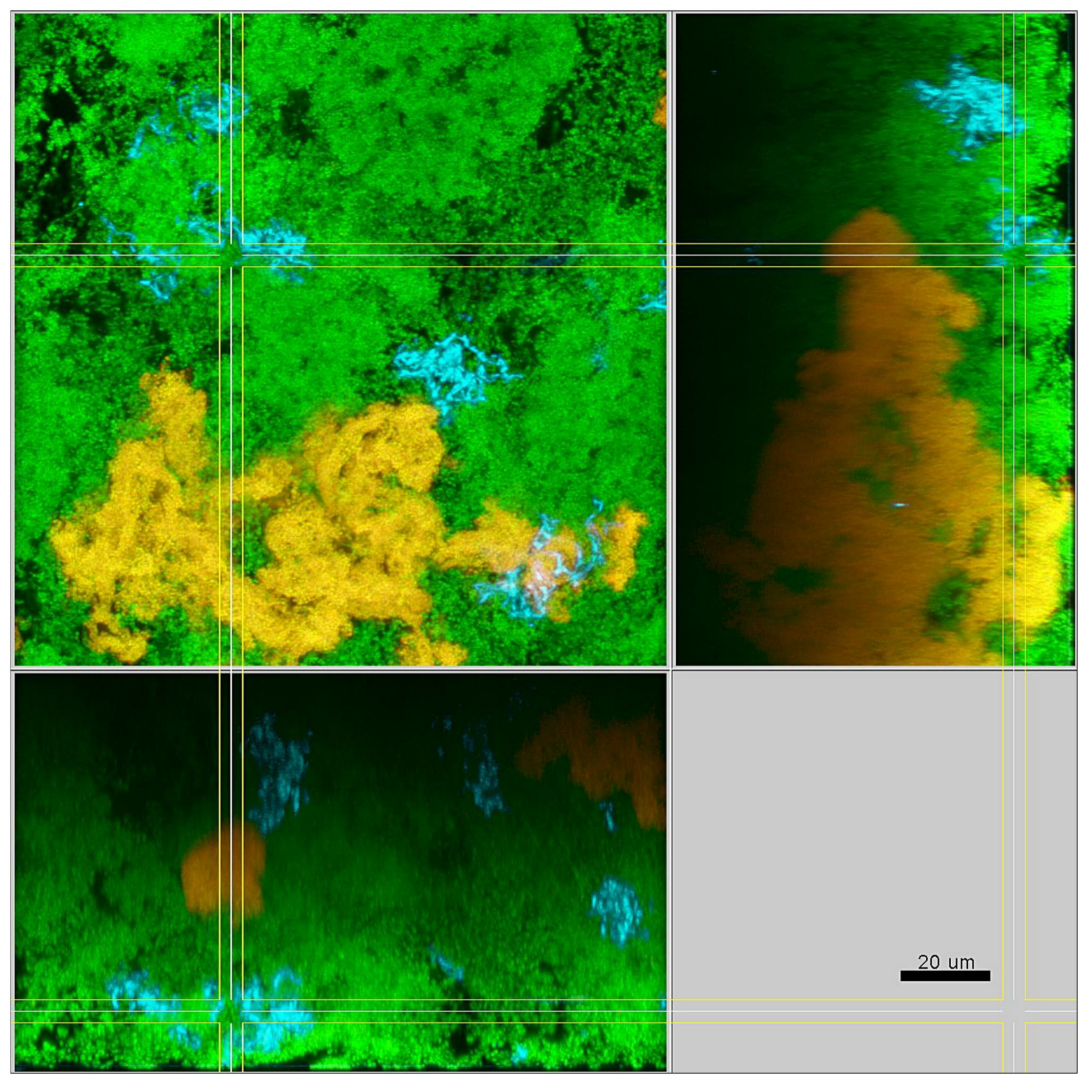
Structure of a control 10-species biofilm after 64.5 h of incubation. Yellow / red: *P*. *intermedia*, cyan: *T*. *forsythia*, green: non-hybridized cells (DNA-staining Yo-Pro-1 + Sytox Green). The yellow colour of *P*. *intermedia* is due to the cross-staining of the L-Pint649-2-Cy3 probe (red) and the universal DNA stains (green).

**Figure 5:**
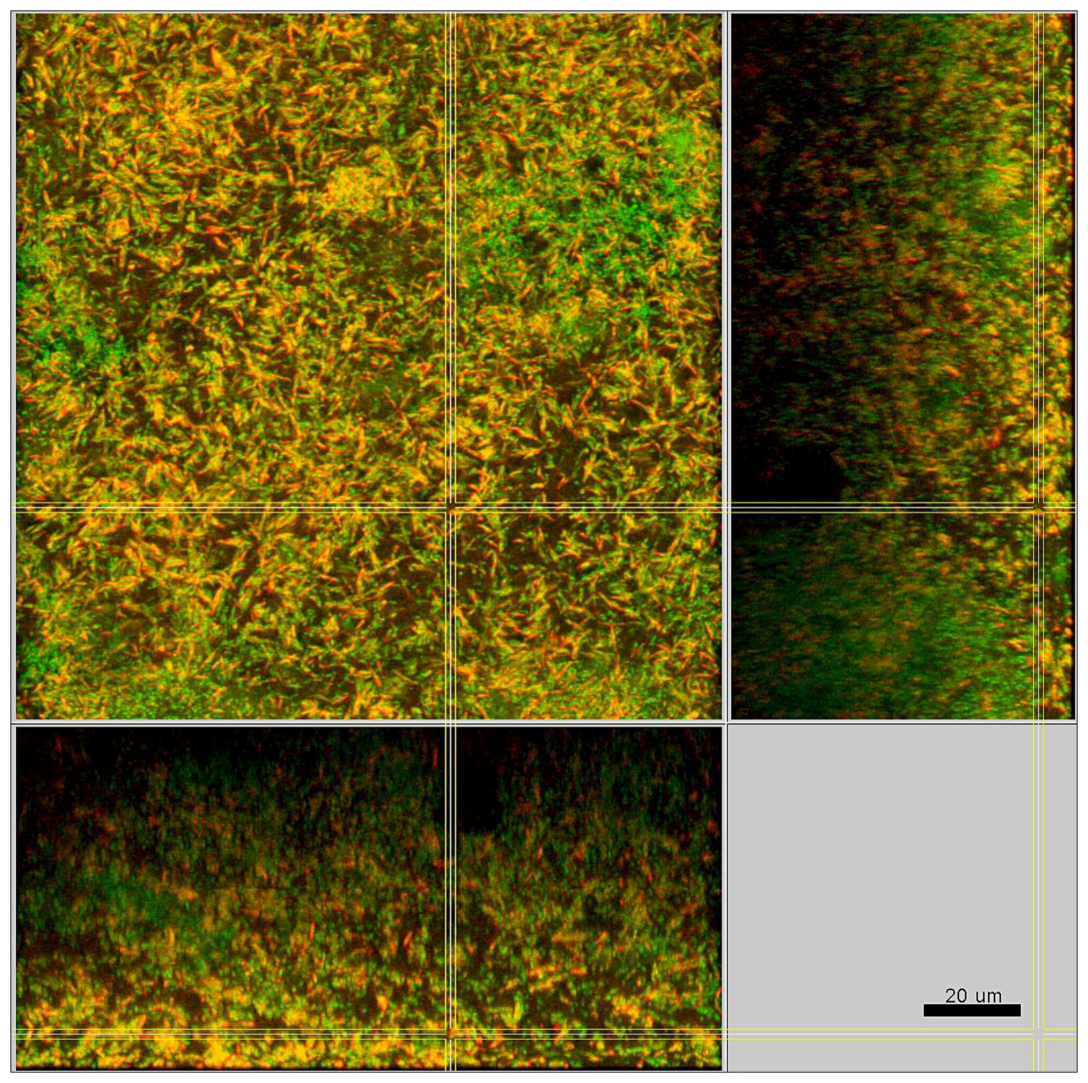
Structure of an 8-species biofilm inoculated without streptococci after 64.5 h of incubation. Yellow / red: *F*. *nucleatum*, green: non-hybridized cells (DNA-staining Yo-Pro-1 + Sytox Green). The yellow colour of *F*. *nucleatum* is due to the cross-staining of the Fnuc133c-Cy3 probe (red) and the universal DNA stains (green).

**Figure 6:**
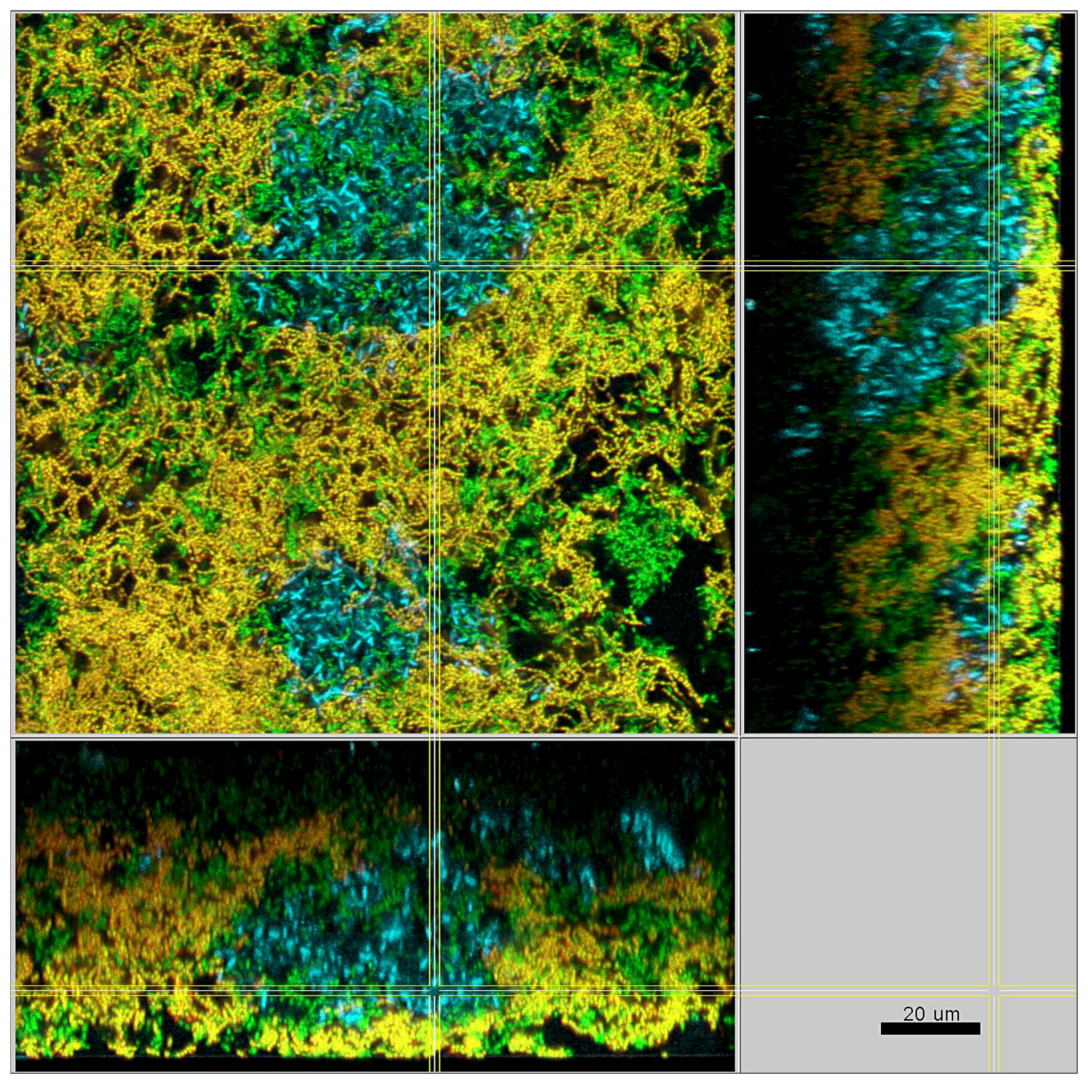
Structure of an 8-species biofilm inoculated without streptococci after 64.5 h of incubation. Yellow / red: *P*. *intermedia*, cyan: *T*. *forsythia*, green: non-hybridized cells (DNA-staining Yo-Pro-1 + Sytox Green). The yellow colour of *P*. *intermedia* is due to the cross-staining of the L-Pint649-2-Cy3 probe (red) and the universal DNA stains (green).

**Figure 7:**
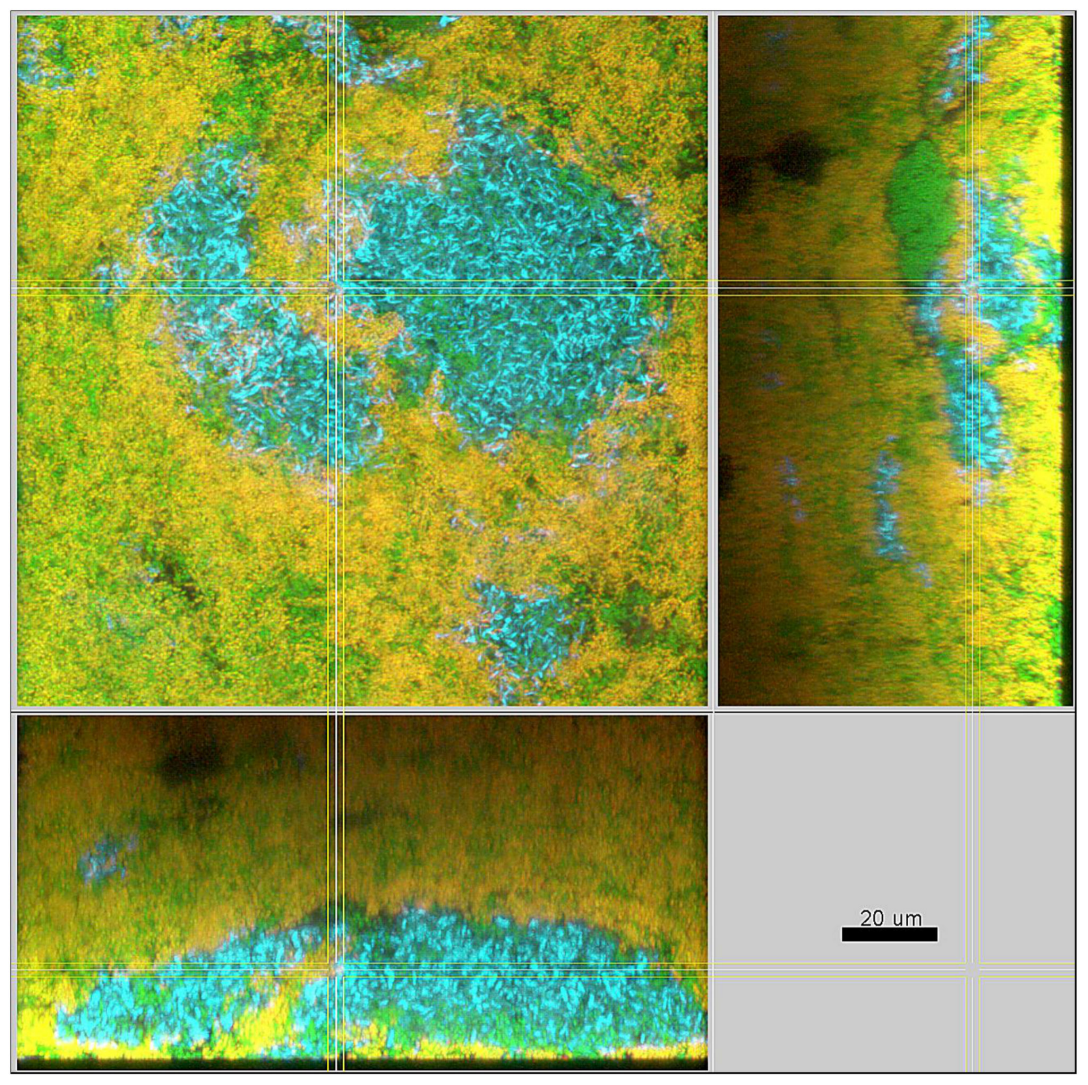
Structure of a 7-species biofilm inoculated without Streptococci and without *A. oris* after 64.5 h of incubation. Yellow / red: *P*. *intermedia*, cyan: *T*. *forsythia*, green: non-hybridized cells (DNA-staining Yo-Pro-1 + Sytox Green). The yellow colour of *P*. *intermedia* is due to the cross-staining of the L-Pint649-2-Cy3 probe (red) and the universal DNA stains (green).

In the presence of streptococci the biofilms were compact with only minimal intercellular spaces and a dense, but defined distribution of the bacteria was apparent (Figure 3). This included the formation of tight bacterial clusters within the biofilms, by several of the involved species, such as *P. intermedia* and *T. forsythia*, as shown in Figure 4. In the absence of streptococci, however, the structure loosened and resulted in a spatially more uniform distribution of all bacteria. In particular, the growth of *F. nucleatum* (Figure 5), as well as *P. intermedia* (Figure 6) in the biofilms appeared much more dispersed than in the control biofilms. Moreover, the absence of streptococci also affected the structural pattern of *T. forsythia* within the biofilms (Figure 6). Remarkably enough, *P. intermedia* showed very distinct morphological changes in the absence of streptococci. In biofilms cultivated without streptococci, P. intermedia bacterial cells were arranged in long chains that morphologically resembled streptococci (Figure 6), while in control biofilms P. intermedia cells were recognized as cocci or short rods (Figure 4). Accordingly, a similar morphology was observed also in biofilms in which the inoculum lacked not only the two streptococci, but also *A. oris*. Hence, the overall the structure of these *A. oris*-free biofilms resembled the one of streptococci-free biofilms, although they did appear more compact (Figure 7).

## Discussion

To initiate a biofilm, bacteria require the ability to attach to a substratum. In the first instance, they attach on the tooth surface, which is coated by a pellicle layer that consists of several salivary glycoproteins [10]. On one hand, the exopolysaccharide matrix plays a crucial role in terms of physical attachment to a biotic or abiotic surface [18]; on the other hand bacteria can adhere via specific receptor interactions. Regarding the attachment strictly from this point of view, streptococci, *A. oris*, and *F. nucleatum* are key biofilm initiators due to their ability to bind directly to the salivary pellicle [10]. Indeed, it has been known since the 1960s that the transition from a healthy flora towards a pathogenic one is mediated by a progression from the early colonizing cocci and short rods to the additional colonisation of fusiform bacteria, finally leading to a dominant incorporation of spirochaetes into the biofilms [5]. However, while receptor binding certainly is crucial in terms of sequential colonisation, biofilm development is also influenced by multiple environmental factors. On one hand, various host-derived factors like pH, salivary IgA titres, the innate immune response to the bacteria, hormone levels, and also the diet will have a significant impact on the composition of the oral microflora [19]. On the other hand, interactions between the biofilm bacteria themselves play a crucial role in shaping the environmental properties. Many of the species colonizing the oral biofilms at a later stage are strict anaerobes, and oxygen-depleting species, such as *F. nucleatum*. These have been shown to play an important role in providing a suitable environment for the late colonizers [20].

The present model system simulates the subgingival conditions only at a nutritional level with serum as the only source of host factors and does not take under consideration any cellular or molecular element of the host immune system. Moreover, it may not directly mirror the *in vivo* conditions in a periodontal pocket with regards to the established gradients for pH, redox potential and host-derived antimicrobial factors. However, despite these technical limitations, the present model is one of the most advanced *in vitro* bofilm models currently available. The use of ten species representative of subgingival plaque provides a complex biofilm setup that allows observations on shifts in the microbial community. In this study, we analysed the consequences on the composition of this *in vitro* biofilm model, once the streptococci (*S. oralis*, *S.* anginosus) alone, or in combination with *A. oris* were omitted. These organisms have long been known as early colonizers in oral biofilms *in vivo* and their role as such was defined both *in vivo* [9,21] and *in vitro* [22]. With regard to the aforementioned prime role of these early colonizers *in vivo*, we expected very little biofilm formation if they were to be omitted from the inoculums, or alternatively, that these biofilms would be inefficiently attached onto the hydroxyapatite discs. In contrast to these expectations, these biofilms developed successfully, reaching similar total counts as biofilms cultivated with streptococci and *A. oris*. They reached a similar thickness and physical resilience in terms of not detaching during the FISH staining procedure, which features plenty of shear-forces. We attribute this finding, at least in part, to the strikingly different morphology and abundance of *P. intermedia*, which appears to majorly contribute to the general structure of these biofilms. Nevertheless, lack of Streptococci and *A. oris* also led to a looser structure and more uniform distribution of the remaining bacteria. Hence, the early colonizers may function in keeping the proximity between all the species within the biofilm.

On the basis of the co-adhesion and adherence capabilities of oral bacteria, as they were defined *in vitro* [10], *F. nucleatum* may be considered the principal organism, other than *A. oris* and the streptococci, capable of direct attachment to the salivary pellicle. Thus, one could hypothesize that it is playing an important role in the initiation of our biofilms. In fact, in an earlier study [12], we found that the concentration of serum used in the growth medium leads to completely different attachment patterns in the early stages of biofilm formation. Without serum in the growth medium, streptococci were the dominant organisms attached to the substratum after 4 h of biofilm formation. However at high concentration of human serum, biofilm formation was delayed and almost no cocci could be detected, as they had been replaced mainly by rods, in all likelihood by *F. nucleatum* [12].

A key finding of this study was that *P. gingivalis* was significantly reduced in biofilms cultivated in the absence of streptococci alone, or in combination with *A. oris*. As the lack of the early colonizing species did not impede biofilm formation in terms of total counts, several factors might have led to this finding. On one hand, streptococci could have played a vital role in facilitating the establishment of *P. gingivalis*, for example by the depletion of environmental oxidants [3]. On the other hand, the strong growth of *P. intermedia* could have hindered the development of *P. gingivalis*, possibly by competition for essential nutrients, such as iron, or by some other antagonistic effects. Iron competition as a cause for the reduced abundance of *P. gingivalis* seems a rather unlikely explanation, since *P. gingivalis* was shown to have a 10-fold higher specific affinity to bind haemin in comparison to *P. intermedia* [23]. However, it is striking that the late addition of streptococci led to an even more pronounced increase of *P. intermedia* compared to the inoculation without streptococci. On the other hand, *P. gingivalis* suffered only a 6.8-fold reduction as a consequence of the late addition of streptococci but declined 20-fold in total absence of streptococci. Thus it seems that, under the conditions of the present experimental model system, the two streptococci are a key factor in facilitating the incorporation of *P. gingivalis* into the subgingival biofilm community.

Another significant finding was the increased growth of *A. oris* in the absence, or after the late addition of streptococci. Earlier experiments had shown that this effect was even more pronounced when the biofilms were grown in serum-free medium. Under these conditions the *A. oris* cell numbers increased by 19-fold in following the addition of the streptococci after 16.5 h of biofilm development (data not shown). These findings partially contrast with a study by Palmer et al. [24] who reported a synergy between *A. oris* and *S. oralis* in terms of biofilm formation, and suggested a metabolic cooperation between these two organisms. However, these authors had used saliva as the sole nutrient and the biofilms consisted of no more than two organisms. In our 10-species model system, a metabolic synergy between *A. oris* and streptococci seems unlikely, as *A. oris* profited from the late addition and, even more pronounced, from the total absence of streptococci. A possible explanation could be competition of *A. oris* and streptococci for binding sites on the salivary pellicle. Both these organisms are known to express the capability of binding protein components on the pellicle [10]. However, in our biofilms the streptococci seemed to outcompete *A. oris*, potentially due to a higher wash-out of this organism during dip-washes. This interpretation is also supported by the finding that with serum-free growth medium, which does not contain any factors capable of inhibiting receptor-mediated adherence to the pellicle, the gain of the streptococci over *A. oris* was most pronounced [12].

While these results are important for the understanding of the basic mechanisms operating in the formation of complex bacterial biofilms, it must be taken into account that the *in vivo* situation inevitably differs to a large extent from any experimental conditions *in vitro*. Nevertheless, the complex environment created with our biofilm model system might contribute substantially to the critical knowledge on the formation of subgingival biofilms. Apart from the evaluation of features like the complex metabolic interplay among the individual species of the biofilm, as is the case in the present study, this model is used for studying the pro-inflammatory action [25-27] or the antimicrobial susceptibility [28-30].

## Conclusions

Biofilms inoculated without *S. oralis*, *S.* anginosus, and *A. oris* developed the same total counts as biofilms inoculated with these *in vivo* early colonizers. Interestingly, the physical resilience of the biofilms without the early colonizers was the same as well. Nevertheless, the absence of early colonizers led to a significant increase in *P. intermedia* cell numbers, along with a significant decrease of *P. gingivalis*. This effect is more likely caused by a synergy between streptococci and *P. gingivalis*, than by a nutritional competition between *P. intermedia* and *P. gingivalis*. Hence, streptococci might possibly facilitate the establishment of *P. gingivalis* into subgingival biofilms. Without early colonizers being inoculated the biofilm structure appeared looser and *P. intermedia* became the most dominant species in the biofilms, appearing in the form of long chains of cocci that morphologically resembled the absent streptococci.

## Supporting Information

Figure S1 **Total counts of bacteria in the biofilms after 64.5 h of incubation.** The boxes represent data from three independent experiments, each performed with triplicate biofilms. Control: All ten species. LateStr: Inoculation without streptococci, addition of streptococci after 16.5 h. NoStr: No streptococci. NoStrNoAori: No streptococci, no *A*. *oris*. No statistically significant differences were detected.
(TIF)
