# Supplementary figures and images for: Impact of Early Colonizers on In Vitro Subgingival Biofilm Formation
Source: PLoS One. 2013 Dec 5;8(12):e83090. doi: 10.1371/journal.pone.0083090 (PMC3855599; doi:10.1371/journal.pone.0083090)

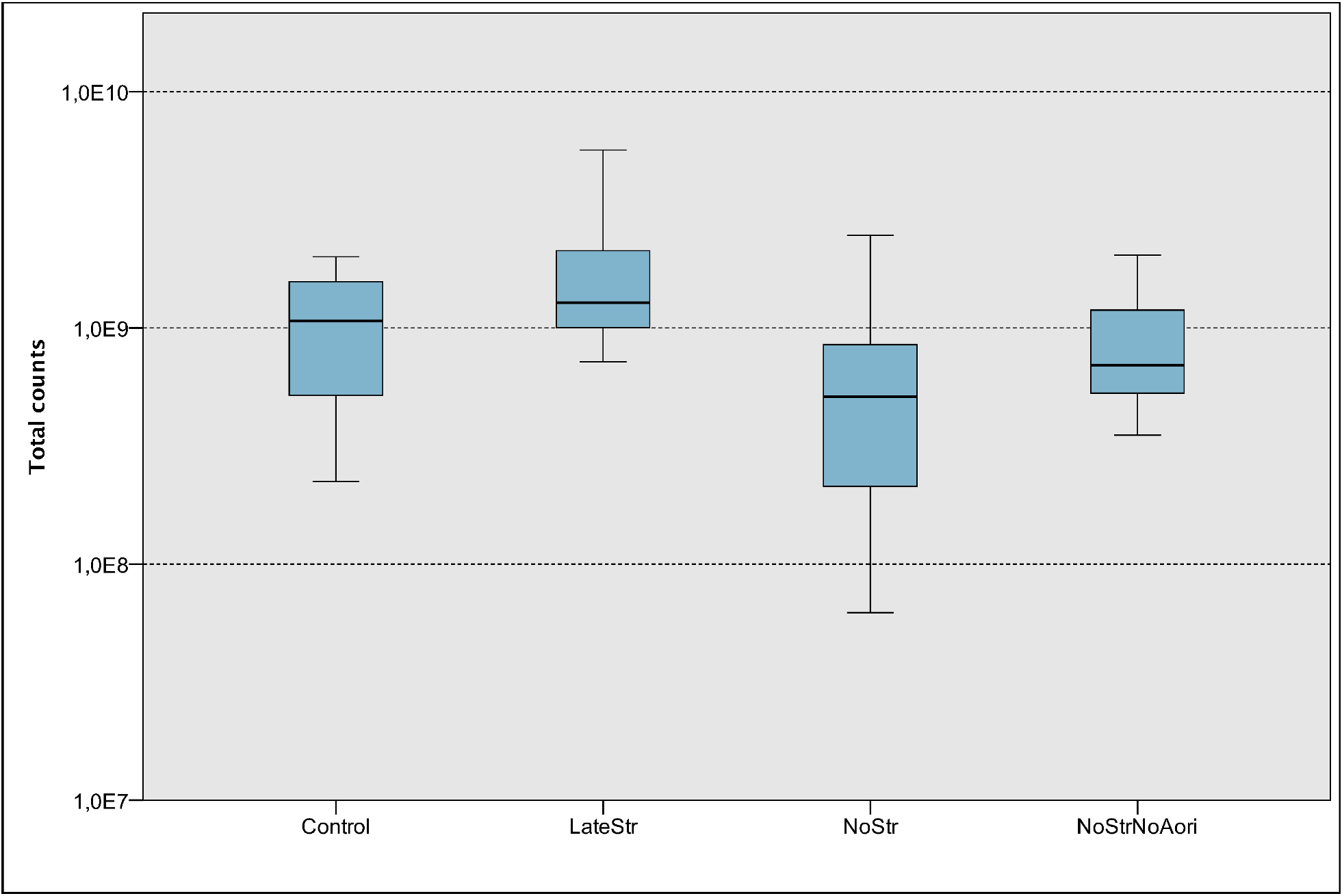

Supplement: Figure S1 — Total counts of bacteria in the biofilms after 64.5 h of incubation. The boxes represent data from three independent experiments, each performed with triplicate biofilms. Control: All ten species. LateStr: Inoculation without streptococci, addition of streptococci after 16.5 h. NoStr: No streptococci. NoStrNoAori: No streptococci, no A. oris. No statistically significant differences were detected. (TIF) [file pone.0083090.s001.tif]
